# Massive functional mapping of a 5′-UTR by saturation mutagenesis, phenotypic sorting and deep sequencing

**DOI:** 10.1093/nar/gkt267

**Published:** 2013-04-22

**Authors:** Erik Holmqvist, Johan Reimegård, E. Gerhart H. Wagner

**Affiliations:** Department of Cell and Molecular Biology, Biomedical Center, Uppsala University, SciLifeLab Uppsala, Box 596, S-75124 Uppsala, Sweden

## Abstract

We present here a method that enables functional screening of large number of mutations in a single experiment through the combination of random mutagenesis, phenotypic cell sorting and high-throughput sequencing. As a test case, we studied post-transcriptional gene regulation of the bacterial *csgD* messenger RNA, which is regulated by a small RNA (sRNA). A 109 bp sequence within the *csgD* 5′-UTR, containing all elements for expression and sRNA-dependent control, was mutagenized close to saturation. We monitored expression from a translational *gfp* fusion and collected fractions of cells with distinct expression levels by fluorescence-activated cell sorting. Deep sequencing of mutant plasmids from cells in different activity-sorted fractions identified functionally important positions in the messenger RNA that impact on intrinsic (translational activity *per se*) and extrinsic (sRNA-based) gene regulation. The results obtained corroborate previously published data. In addition to pinpointing nucleotide positions that change expression levels, our approach also reveals mutations that are silent in terms of gene expression and/or regulation. This method provides a simple and informative tool for studies of regulatory sequences in RNA, in particular addressing RNA structure–function relationships (e.g. sRNA-mediated control, riboswitch elements). However, slight protocol modifications also permit mapping of functional DNA elements and functionally important regions in proteins.

## INTRODUCTION

Forward and reverse genetics methods are valuable tools to link phenotypes to DNA sequences. Forward genetics identifies nucleotide changes that cause a phenotypic change, and reverse genetics identifies the phenotype associated with a particular mutation. In reverse genetics, site-directed mutagenesis can be used to, for example, pinpoint nucleotides in DNA/RNA sequences at which regulators bind and can assess RNA structure–function relationships [e.g. (1,2)]. Random mutagenesis by polymerase chain reaction (PCR) under error-prone conditions is a powerful method for creating large pools of mutants (3). For instance, error-prone PCR followed by fluorescence-activated cell sorting (FACS) analysis has been used to generate variants of the green fluorescent protein (GFP) with increased intensity and more efficient folding (4). Related to the work presented here, error-prone PCR followed by phenotypic screening has identified base changes that affect expression and stability of the small RNA (sRNA) MicA, as well as MicA-dependent post-transcriptional regulation (5). Even though such approaches have turned out to be successful in identifying functionally important nucleotides, they are tedious and suffer from low throughput, as each mutant needs to be phenotypically assayed one by one.

To increase throughput in reverse genetics, several recent articles have described methods for scoring effects on gene expression from large numbers of sequence variants. The RNA-ID method was designed to study *cis*-regulatory RNA sequences in yeast; short random sequences were inserted into an messenger RNA (mRNA), and effects on translation efficiency were monitored by FACS of fluorescent protein expression (6). Kudla *et al.* (7) used a library of 154 synthetic variants of *gfp* to study gene expression changes arising from synonymous mutations. Another article reported on a multiple mutation-, FACS- and high-throughput-sequencing method used for mapping protein binding and its energetics in *transcriptional* regulation (8). Two additional publications also describe similar methods for transcriptional regulation (9,10) and use mRNA abundance measurements as readout for gene expression. However, changes in DNA sequences that involve insertions (6) may introduce unwanted effects arising from different sequence lengths of the analyzed variants. Additionally, mRNA abundance does not always accurately report on a mutation’s effect on gene expression, as mRNA and protein levels are not always correlated, and protein expression often is predominantly regulated at the post-transcriptional level (9,11). For instance, studies of several bacterial mRNAs showed that sRNA-mediated regulation can give altered protein levels without significantly affecting mRNA levels (12,13).

For functional screening and mapping of high numbers of mutations in single experiments, we present here a method that combines saturation mutagenesis, phenotypic cell sorting and high-throughput sequencing. This method is particularly powerful for studies of post-transcriptional regulation, but it is easily adaptable for studies of transcriptional regulation as well. Our method does not rely on insertion of sequences but generates nucleotide substitutions, eliminating the risk of unwanted effects through changes of sequence length.

In bacteria such as *Escherichia coli*, small regulatory RNAs play a major role as post-transcriptional regulators of gene expression [reviewed in (14)]. By base pairing to complementary sequences in target mRNAs, sRNAs affect target mRNA translation, target mRNA stability or both (14). In most cases, sRNA-regulation relies on help by the RNA-binding protein Hfq, which binds to both sRNAs and target mRNAs to accelerate their interaction [e.g. (15)]. In addition, many sRNAs are severely destabilized in the absence of functional Hfq (12,16). We have recently reported on the Hfq-dependent regulation of CsgD protein expression by the two sequence-related sRNAs OmrA and OmrB (12). Binding of these sRNAs to the 5′-untranslated region (5′-UTR) of the *csgD* mRNA inhibits translation of CsgD, a transcription factor involved in biofilm formation.

To test the method described in this article, we have monitored expression from a large number of mutants of *csgD* in the absence or presence of OmrA. The results presented here demonstrate the strength of the method in defining sequence features important for translation and/or stability of the selected model mRNA, as well as delineating the sequences involved in sRNA-mediated regulation.

## MATERIALS AND METHODS

### Chemicals, reagents and oligodeoxyribonucleotides

All chemicals and reagents used in this study were purchased from Sigma-Aldrich or Fermentas unless otherwise specified. Oligodeoxyribonucleotides were purchased from Sigma-Genosys or Metabion and are listed in Supplementary Table S1.

### Strains and growth conditions

*Escherichia coli* TOP10 cells (Invitrogen) were grown aerobically in Luria broth (LB) at 37°C. Growth was spectrophotometrically monitored by measuring optical density at 600 nm. When appropriate, the growth medium was supplemented with ampicillin (100 µg/ml) and chloramphenicol (30 µg/ml).

### Cloning

To construct the translational fusion plasmid pCsgD::GFP, a DNA fragment consisting of the pLtet-O-1 driven translational *csgD-gfp* fusion was amplified from plasmid pEH87 (12) by PCR using primers EHO-560/EHO-561 (containing *Hae*II restriction sites). The resulting fragment was cut with *Hae*II and ligated into *Hae*II-digested pSU2719 (17). To construct pOmrA, pUC19 was linearized by PCR (primers EHO-570/EHO-571, containing *Eco*RI and *Bam*HI sites, respectively), cut with *Eco*RI/*Bam*HI and ligated to an *Eco*RI/*Bam*HI-cleaved PCR product amplified from plasmid pEH67 (12) (primers EHO-572/EHO-573) containing the P_LlacO_ promoter sequence followed by the *omrA* sequence.

### Error-prone PCR

Error-prone PCR on plasmid pCsgD::GFP was carried out in a two-step PCR reaction using the GeneMorph II EZClone Domain Mutagenesis Kit (#200552, Stratagene). The first PCR reaction (Mutant Megaprimer Synthesis) contained Mutazyme II reaction buffer, dNTPs (0.2 mM each), 0.2 µM each of primers EHO-522 and EHO-575, 1 ng of plasmid pCsgD::GFP and 1.25 units of Mutazyme II DNA polymerase in a total volume of 25 µl. PCR program: initial denaturation step at 95°C for 2 min, then 30 cycles of a 30-s denaturation step at 95°C, 30-s annealing step at 52°C and 60-s elongation step at 72°C. The PCR reaction was finalized by 10 min elongation at 72°C. The PCR product was analyzed on a 1% agarose gel and purified with the PCR Purification Kit (QIAGEN). The second PCR (EZClone reaction) contained EZClone enzyme mix, 12.5 ng of plasmid pCsgD::GFP, 125 ng of the PCR product from the first PCR reaction (used as primers in this reaction) and EZClone solution in a total volume of 25 µl. Second PCR program: initial denaturation at 95°C followed by 25 cycles of a 50-s denaturation step at 95°C, 50-s annealing step at 60°C and 6 min elongation step at 68°C. After 2 min on ice, 1 µl of *Dpn*I was added for 2 h at 37°C to remove the template plasmid.

### Transformation

*Escherichia coli* TOP10 cells harboring either pUC19 or pOmrA were made competent by washing exponentially growing cultures three times in 10% glycerol. In all, 50 µl of competent cells were subsequentially transformed with 2 µl from the EZClone reaction by electroporation. After recovery for 1 h at 37°C in 1 ml of LB medium, cells were spread on agar plates supplemented with ampicillin (50 µg/ml) and chloramphenicol (30 µg/ml) and incubated o/n at 37°C.

### FACS

Ten thousand colonies obtained after transformation of the mutant plasmid library were washed off the agar plates with LB medium, pooled, divided in triplicates, diluted in LB containing ampicillin and chloramphenicol and grown in Erlenmeyer flasks o/n at 37°C with rotation at 200 rpm. The o/n cultures were diluted 100-fold in fresh medium with antibiotics and grown at 37°C at 200 rpm. When cultures reached an OD_600_ of 0.2, 5 ml of each culture was withdrawn and pelleted by a 10-min centrifugation at 4000 rpm. Cell pellets were resuspended in 10 ml of sterile phosphate buffered saline. FACS was carried out using a BD FACSAria cell sorter, and data analysis was done using the FlowJo software. Sorted cells (≈10^6^ cells/ pool) were pelleted by centrifugation at 13 000 rpm for 10 min and resuspended in 20 µl of sterile H_2_O.

### High-throughput DNA sequencing

Each pool of sorted cells was used as template in a PCR reaction to generate sequences to be analyzed by deep sequencing. Before PCR, sorted cells were denatured for 5 min at 95°C. Each PCR reaction contained 2 µl sorted and denatured cells, dNTPs (0.2 mM each), 0.4 µM each of primers EHO-522 and EHO-575, HF PCR Buffer, Phusion High-Fidelity DNA Polymerase (Finnzymes) and sterile H_2_O in a total volume of 50 µl. PCR program: an initial denaturation step at 98°C, 30 cycles with a 10-s denaturation step at 98°C, a 10-s annealing step at 64°C and a 15-s elongation step at 72°C followed by a final 5 min elongation step at 72°C. PCR products were analyzed on a 1% agarose gel and purified with the PCR Purification Kit (QIAGEN).

Sequencing libraries were prepared from 0.6 µg of PCR product according to the TruSeq DNA sample preparation guide #15005180 revC using reagents from the TruSeq DNA sample prep kit set A and set B v2 (Illumina). Briefly, the DNA fragments were end repaired followed by purification using AMPure XP beads (Beckman Coulter). One A base was added to the blunt ends of the DNA fragments and adapters, and index tags for sequencing were ligated, followed by purification using AMPure XP beads. The DNA fragments and the library were size selected on a 2% agarose gel, and the fraction containing the 285 bp adapter-ligated fragments was excised from the gel, purified using a QIAGEN gel extraction column (Qiagen) and PCR amplified for 10 cycles, followed by purification using AMPure XP beads (Beckman Coulter). The quality of the library was evaluated using the Agilent Technologies 2100 Bioanalyzer and a DNA 1000-kit. The adapter-ligated fragments were quantified by quantitative PCR (qPCR) using the Library quantification kit for Illumina (KAPA Biosystems) on a StepOnePlus instrument (Applied Biosystems/Life technologies) before cluster generation and sequencing.

A 16 pM solution of DNA libraries in equimolar amounts was subjected to cluster generation on the cBot instrument (Illumina Inc.) using the TruSeq PE cluster kit v3. Paired-end sequencing was performed for 100 cycles in a HiSeq2000 instrument (Illumina Inc.) using TruSeq SBS chemistry v3, according to the manufacturer’s protocols. Base calling was done on the instrument by RTA 1.13.48, and the resulting .bcl files were converted to Illumina qseq format with tools provided by OLB-1.9.0 (Illumina Inc.). To separate samples and the PhiX control DNA sequenced in the same lane as the sample libraries, the qseq-files were de-multiplexed, allowing for one mismatch. De-multiplexing was done with CASAVA 1.7.0 (Illumina Inc.). Additional statistics on sequence quality were compiled from the base call files with an in-house script.

### Site-directed mutagenesis

Site-directed mutagenesis was carried out using the Quick-Change II kit (Stratagene). Mutagenic primers are listed in Supplementary Table S1.

### Fluorescence measurements in 96-well plates

Growth and fluorescence monitoring in 96-well plates was done as described in (13).

### Data analysis

Illumina reads were trimmed with cutAdapt (v0.9.5) (18) with a phred quality filter 30 (−q 61) to remove reads of low quality. Read pairs were merged into one read using SeqPrep (http://seqanswers.com/wiki/SeqPrep) with at least 30 bp overlap (−o 30). All reads of incorrect length, i.e*.* sequences with insertion, deletions or those not properly merged, were removed from further analysis. All remaining sequences were sorted based on how many mutations they contained, the nucleotide number(s) within the sequence and on the identity of the nucleotide change. Only reads with one mutation, a total of 474 (3 × 158), were kept for further analysis. Heatmap and dendogram of single mutation distribution was generated with heatmap.2 in the gplot library in R. Differences in abundance for all single mutation between two different conditions were analyzed using DEseq (19).

## RESULTS

### Description of the method

**Figure 1.**
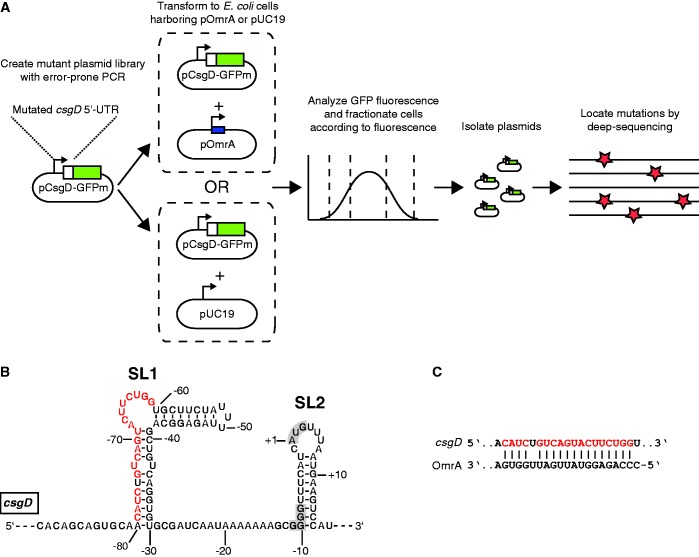
Method outline and model system (**A**) Flow chart of the mutagenesis and cell sorting strategy. (**B**) Sequence and secondary structure of the *csgD* mRNA as deduced from structure probing experiments and phylogenetic conservation (12). Red nucleotides indicate the binding sequence for the sRNA OmrA. The SD sequence and the start codon are shaded in gray. The structural elements SL1 and SL2 are indicated. (**C**) Base pairing between *csgD* mRNA and OmrA. Red indicates the binding sequence for OmrA on *csgD* mRNA, as in (B).

The aim of this study was to establish a method that, in a single experiment, can associate all possible mutations in a selected sequence with changes in gene expression. The experimental setup of the described method is as follows (Figure 1A). First, a gene of interest is translationally fused to the *gfp+* allele on a plasmid. Mutations are introduced by error-prone PCR, and the mutant plasmid library is transformed into *E. coli* cells. The resulting transformants are pooled and subjected to single-cell fractionation with respect to distinct fluorescence levels using FACS. Finally, the mutant sequences present in each fraction are PCR amplified and subjected to high-throughput sequencing.

To test the method, we chose to analyze the effects of nearly all possible mutations in a 109 bp long sequence on the translational activity of the *csgD* mRNA in *E. coli*. This mRNA is well suited for this purpose because its translational activity is controlled both extrinsically and intrinsically. Expression of *csgD* is highly regulated, both at the transcriptional and post-transcriptional level. More than 10 transcription factors have been shown to bind at the *csgD* promoter and affect *csgD* transcription (20–24). In addition, at least four sRNAs bind to the *csgD* mRNA to inhibit translation (12,25–27). Our previous work has demonstrated that the 5′UTR of the *csgD* mRNA has two conserved structure elements, stem-loop 1 and 2 (SL1 and SL2, Figure 1B). SL1 harbors a binding site for OmrA, an sRNA that inhibits translation of CsgD, and SL2 is a stem-loop that partially inhibits translation by sequestering the Shine-Dalgarno (SD) sequence [Figure 1B and C, (12)]. Thus, mutations that interfere with sRNA binding in SL1 (interaction sequence, Figure 1C), or destabilize the SL2 structure, are expected to increase gene expression.

**Figure 2.**
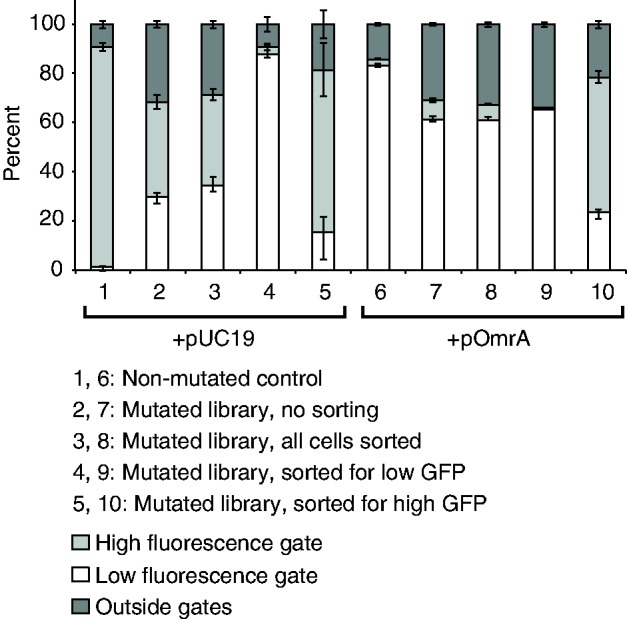
Statistics on FACS analysis. Diagram showing the percentage of cells within the gates set as high or low GFP fluorescence, as well as cells outside of the gates, in unsorted samples and samples sorted for all cells, high fluorescence or low fluorescence. The fluorescence distribution of cells having the wild-type plasmid pCsgD-GFP in combination with either pUC19 or pOmrA is also shown. Error bars represent the standard deviations from three replicate experiments.

Accordingly, gene expression was monitored by GFP fluorescence from cells carrying the translational fusion plasmid pCsgD::GFP. In this plasmid, the constitutive P_LtetO_ promoter drives expression of the *csgD* 5′-UTR, which is translationally fused to the *gfp+* allele at codon 15 of *csgD*. To map functionally important nucleotides that either affect translational activity *per se* or sRNA-dependent repression, we mutagenized the 5′-UTR of the *csgD* mRNA on pCsgD::GFP close to saturation and monitored effects of each mutation on translational activity. The mutant plasmid library was constructed by error-prone PCR of a selected DNA fragment in the *csgD* 5-UTR (109 bp, containing all elements necessary for translation and OmrA-mediated regulation), which was re-inserted into pCsgD::GFP. This library (pCsgD::GFPm) was transformed into *E. coli* cells that harbored either an OmrA overexpression plasmid (pOmrA) or a control vector (pUC19). The resulting transformants were pooled and sorted according to fluorescence by FACS (in triplicates), where gates for sorting were set according to the fluorescence levels in strains with the wild-type pCsgD::GFP plasmid in cells harboring either pOmrA or pUC19. The percentage of cells with high or low fluorescence in each sample before and after sorting is shown in Figure 2.

### Data analysis

**Figure 3.**
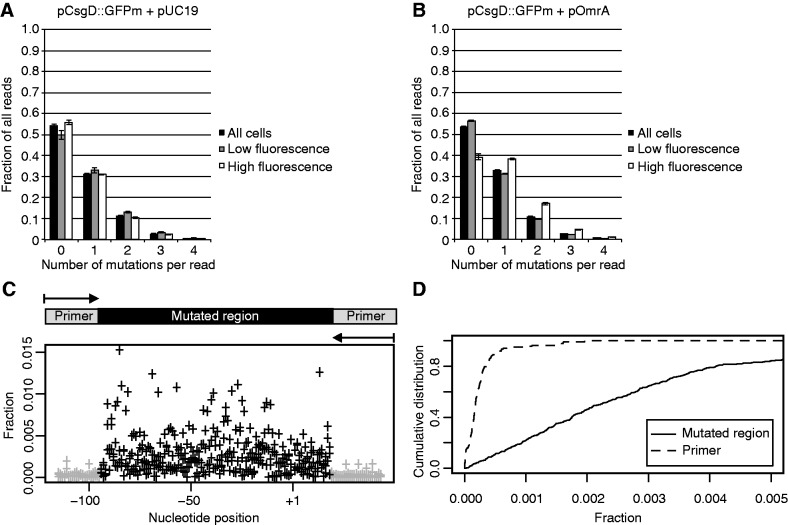
Distribution of mutations. (**A**) and (**B**) Fraction of obtained reads with 0, 1, 2, 3 or 4 mutations in the indicated samples. Error bars show the standard deviation from three replicate experiments. (**C**) The mean fraction of each single mutation in the unsorted sample based on three biological replicates. The fraction of each mutation was calculated by dividing the number of reads of that mutation by the total number of reads with a single mutation. Gray crosses represent mutations mapping to the primer region, and black crosses represent mutations within the mutated region. The schematic on top of the graph indicates the region that has been sequenced and analyzed for mutations. (**D**) Cumulative distribution of the mutation fraction pattern shown in (C) divided into the two subgroups with mutations in the primer regions (black dashed line) and the mutated region (black continuous line).

After FACS sorting, sequences encompassing the mutated regions in pooled extracted plasmids were PCR amplified and analyzed by high-throughput sequencing. After merging and removing low-quality reads, all individual sequences remaining in the library were identified. Most reads in all samples had wild-type sequence. Of the mutant sequences, most had one mutation (30–40%) and <5% had more than three (Figure 3A and B). All sequences with single mutations showed 90% sensitivity and 90% specificity for the mutated region, compared with the adjacent primer sequences (Figure 3C and D).

**Figure 4.**
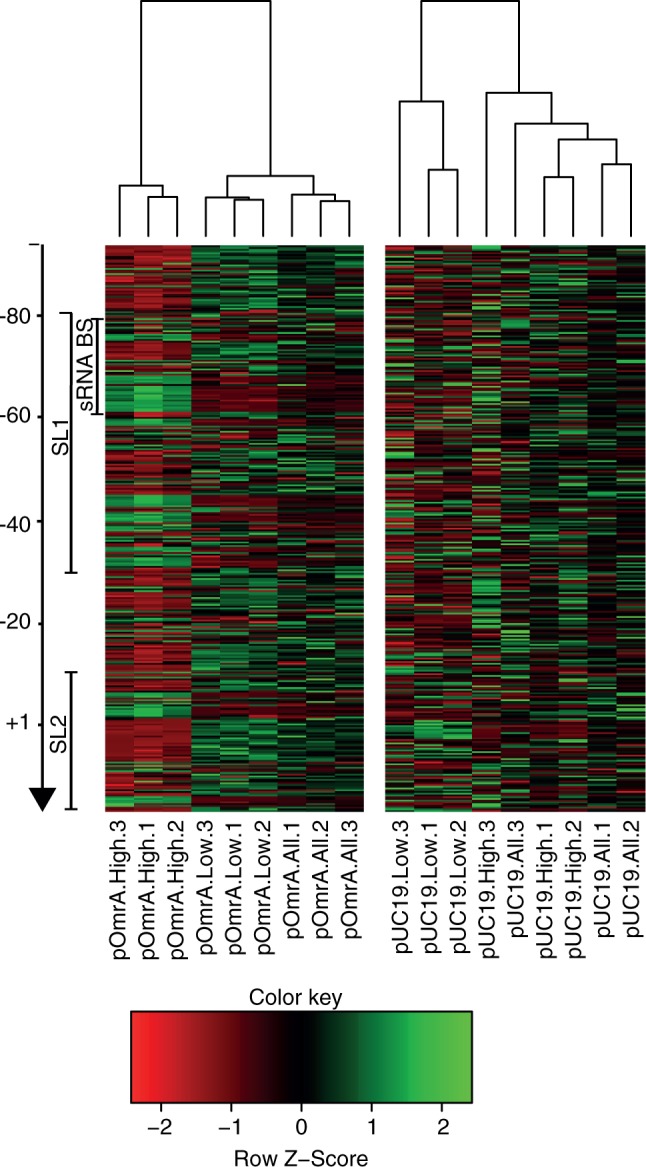
Differences in mutation pattern between different samples. Each column in the heatmap represents the mutation pattern of one sample. Each sample is named according to the plasmid it carried (pOmrA or pUC19), which bin it was sorted into (high, low or all) and which biological replicate it represents (1, 2 or 3). The dendogram on top of the heatmap shows the hierarchical clustering of similarities in the mutation patterns between the different samples. Each row in the heatmap represents one mutation, e.g U-75A, and each cell is colored from red (under-represented compared with the other cells in the same row) to green (over-represented compared with the other cells in the same row). Both stem-loops (SL1 and SL2) and the sRNA binding region (sRNA BS) are indicated by bars on the left.

To assure that the read counts of the different mutations correlate with the FACS sorting, we clustered the samples based on their single mutations patterns. For most of the samples, the biological replicates for the different conditions clustered in the dendogram (Figure 4). This indicates that the variation in read counts of the different mutations between the samples depends on the FACS sorting condition and is not caused by other technical issues. The most distinct mutation pattern, i.e the pattern that differed the most from the other patterns, was found in high-fluorescence samples in which OmrA was overexpressed; samples from unsorted cells and those with wild-type levels of fluorescence showed a more similar mutation pattern, indicating that most mutations were phenotypically silent (Figure 4). Compared with unsorted cells, significantly over- or under-represented single mutations in each pool of triplicates were identified by DEseq (19). The variance of three biological replicates for each condition, the difference ‘between’ conditions and the total mutation count determines the probability to obtain this difference by chance. Finally, *P*-values were adjusted based on the multiple testing problem, and all mutations with a *P* value of <0.05 were kept for further analysis.

### Mutations in SL1

**Figure 5.**
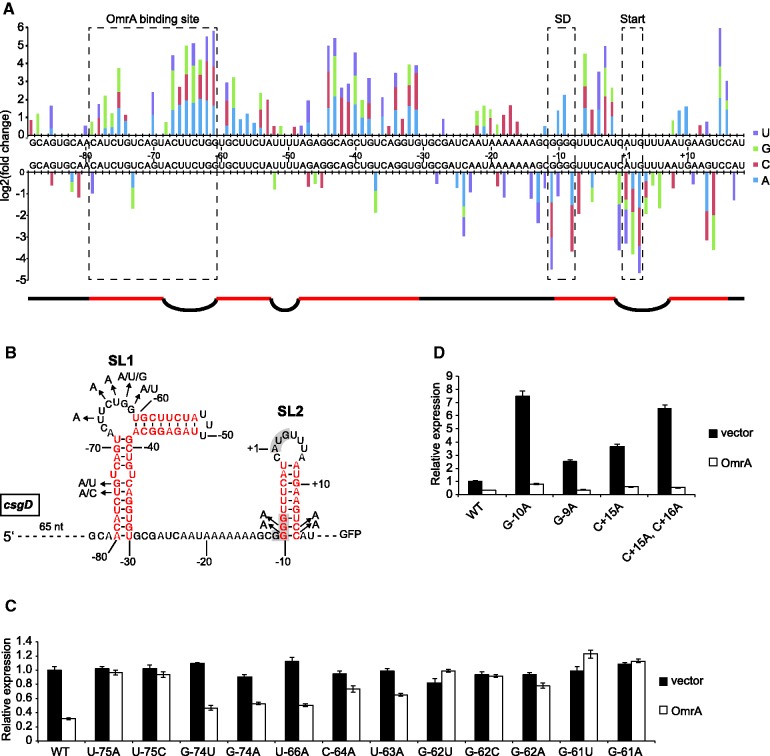
Mutations associated with elevated *csgD-gfp* expression. (**A**) Significantly under- and over-represented mutations in samples that were sorted for high fluorescence in the presence of OmrA compared with samples sorted for all fluorescence levels. Mutations were identified by Illumina sequencing. The RNA sequence of the *csgD* 5′-UTR is shown in the middle of the graph in 5′–3′ direction. Numbers next to the sequence indicate nucleotide positions relative to A in the AUG start codon (set as +1). The OmrA binding site, the SD sequence and the start codon are indicated by dashed boxes. A schematic under the graph represents the secondary structure of the *csgD* 5′-UTR where black lines and bulges indicate unpaired, and red lines indicate paired nucleotides [same coloring as in (**B**)]. (B) Sequence and secondary structure of the 5′-UTR of *csgD* mRNA. Arrows indicate mutations that where re-introduced and functionally assayed [see (**C**) and (**D**)]). Coloring and numbering as in (A). (C) and (D) Fluorescence measurements from cells with pCsgD::GFP with re-introduced mutations selected from the Illumina sequencing data set.

Mutations associated with loss of OmrA-mediated regulation were expected in cells that despite the presence of the repressing sRNA displayed high GFP levels. Indeed, mutations within the sRNA binding site (−79 to −61, Figure 5A and B) were highly enriched in high fluorescence samples compared with unsorted samples (Figure 5A). Within this region, the bulged nucleotides at which OmrA binding initiates were most enriched (−61 to −67, Figure 5A and B). To evaluate the biological significance of the obtained mutations, highly enriched mutations were individually re-introduced into plasmid pCsgD::GFP by site-directed mutagenesis. Fluorescence measurement from these constructs confirmed the loss of OmrA-dependent regulation for mutations in the binding site, whereas fluorescence levels in the absence of OmrA were unchanged (Figure 5C). Thus, these mutations do not affect translation rates *per se* but cause insensitivity to OmrA.

Interestingly, almost all enriched mutations in SL1 outside of the sRNA binding site (47/51) disrupt intramolecular base pairing (Figure 5A and B), suggesting that correct folding and structural stability of SL1 (in both helical elements, Figure 5B) is a determinant for efficient sRNA regulation. Structure-constrained presentation of initiation sequences in a loop/bulge is known to promote rapid binding of antisense RNAs (28).

### Mutations in SL2

In contrast to mutations that entail loss of sRNA regulation, higher fluorescence in cells that overexpress OmrA may also reflect increased translation rate *per se*, e.g. by mutations that increase expression of both the active and repressed state. The SD sequence of *csgD* is located within the stable SL2 (Figure 5B) whose destabilization increases translation (12). Accordingly, SL2-destabilizing mutations were enriched in samples sorted for high fluorescence in the presence of OmrA (Figure 5A). Higher expression owing to mutations in SL2 does not seem to rely on loss of sRNA regulation, as OmrA can regulate these mutants when re-created by site-directed mutagenesis (Figure 5D). In general, mutations in SL2 that confer higher fluorescence disrupt base pairs, whereas under-represented mutations create stronger base-pairs (Figure 5A and B). The opposite is seen on sorting for lower GFP expression in the absence of OmrA, i.e. over-representation of mutations that create stronger base pairs and under-representation of those that break base pairs (Supplementary Figure S1). Also, mutations in the AUG start codon were strongly under-represented when sorting for higher fluorescence but over-represented in the low fluorescence pool (Figure 5A and Supplementary Figure S1). Mutations that change the wild-type SD sequence (GGGG) to the optimal GAGG or GGAG gave higher fluorescence, whereas changes to poorly matching sequences (AGGG, UGGG, CGGG, GUGG, GGGA, or GGGC) were under-represented, even though several of these disrupt base pairs in SL2, which otherwise increases translation (Figure 5A).

### Possible limitations of the method and additional clarifications

**Figure 6.**
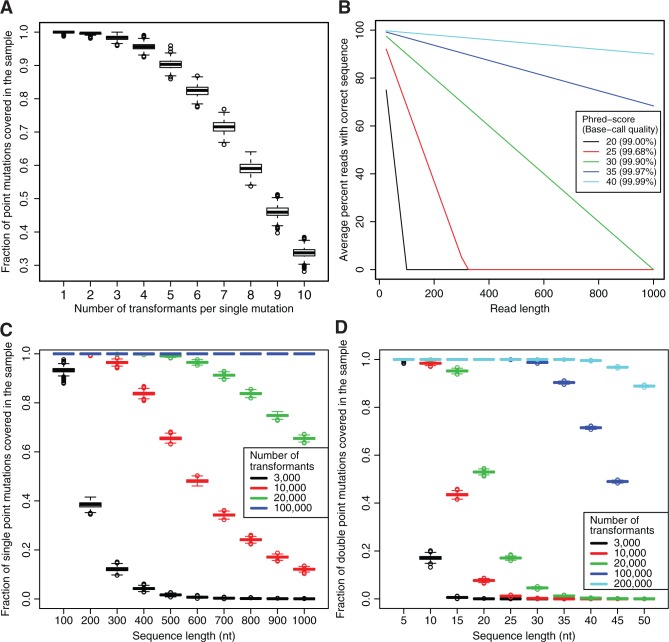
Mutation distribution simulations. (**A**) Simulated data of 1000 runs on single mutation random mutagenesis of 3000 transformants with a 109 nt sequence represented with boxplots. Each boxplot represents the distribution of the fractions of single mutations covered (*y*-axis) as a function of the number of times it has to occur (*x*-axis). (**B**) Average percentage reads of correct sequence depending on different Phred-scores (base call accuracy in parentheses). (**C**) Simulated data of 1000 runs on single mutation random mutagenesis. Each boxplot represents the distribution of the fractions of single mutations covered (*y*-axis) at least five times as a function of sequence length (*x*-axis). The different colors represent different numbers of single mutation transformants. (**D**) Simulated data of 1000 runs on double mutation random mutagenesis, plotted as in (C), but all symbols here refer to double mutation transformants.

Three potential limitations of our method need to be discussed. The first concerns the initial number of transformants needed to cover all mutations in the sequence of interest. Assuming random mutagenesis, simulated data suggest that at 99.5% probability 3000 transformants with single mutations should be sufficient to cover each mutation at least once, and that on average, 90% of the mutations would be covered five times (Figure 6A). The second important issue concerns that the chosen high-throughput sequencing method should produce reads that cover the entire region with high base calling at sufficient depth (Figure 6B). In each of our experiments, we obtained at least 2 million reads with a phred score cut-off of 30, i.e. a base call accuracy of >99.9%, suggesting ≥80% of our reads should have the same sequence as the sequenced DNA molecule. Assuming that wrong base calls are independent or systematic between experiments, our coverage of ≥2000 reads per cell should be sufficient to amplify the true signal above the noise. In practice, we recovered >90% of the mutations with sufficient specificity. Third, an additional possible limitation lies in the fitness cost that can be associated with overproduction of a fluorescent protein at high levels. We tested this by sorting mutants associated with high levels of GFP expression and could confirm that these mutants were under strong counter selection (data not shown). Thus, it is of great importance to optimize the ratio of expression levels so that all populations one aims at analyzing are present in the culture used for sorting.

Although this study presents data for sequences with single point mutations, an extended study could also include an analysis of sequences with two mutations. A combined analysis of single versus double mutations is expected to identify sequence positions that individually alter the phenotype but in combination restore wild-type-like behavior. This is particularly powerful in identifying RNA structure motifs, e.g. reflecting disruption and restoration of intramolecular base pairing. The limiting factor for an analysis of double-mutation data sets is the need for correspondingly high numbers of transformants. Although the need for transformants in single mutation analysis is linear with sequence length, it is quadratic for double mutations (cf. Figure 6C and D). There are also limitations in the length of the sequence that can be conveniently analyzed by this method. For longer sequences, when analyzing single point mutations, the number of transformants is not limiting, but the requirement for long enough high-quality reads is. Even at a 99.9% base call accuracy, 50% of all reads may be incorrect in a 500 nt long sequence (Figure 6B). This asks for an increase in sequencing depth to handle the smaller signal-to-noise ratio. With improved methods that improve sequence read quality and increase read length, this method will be applicable to both longer sequences and double-versus-single mutation analysis.

## DISCUSSION

This study describes a new method for massively parallel mutational analysis in one experiment. By combining saturation mutagenesis, phenotypic cell sorting and high-throughput DNA sequencing, almost all possible point mutations in a 109 bp DNA sequence encoding the 5′UTR of a bacterial mRNA were linked to translational activity and to susceptibility to regulation by an sRNA. In contrast to classical genetics, this method reveals not only positions crucial for gene regulation or expression but also identifies all phenotypically silent mutations and thus is a powerful tool for in-depth mapping of sequence–function relationships in any RNA of interest. For example, *cis-*acting elements such as riboswitches can be analyzed without substantial changes in our protocol. This method can also be used to identify functionally important residues in DNA elements or proteins. Depending on the question at hand, FACS based on GFP signals can be substituted by other suitable phenotypic sorting methods.

We have tested this method by mutagenizing the 5′UTR of the *csgD* mRNA and monitored fluorescence from a mutant library of a *csgD-gfp* translational fusion. In accordance with previous work (12), the method successfully identified regions within the mRNA that are important for translation. Mutations in SL1 that weaken or abolish the interaction with the sRNA OmrA, as well as mutations that destabilize SL2, led to increased GFP levels, underscoring the importance of these elements for *csgD* expression. In contrast to the expected mutations in SL1 and SL2, the data revealed mutations that are not easily explained by our current knowledge on *csgD* expression. Tentative speculations on the reason for enrichment of these mutations might be effects on mRNA stability or changes in a putative Hfq-binding site. In addition, some of these mutations might reflect changes in long-range secondary structure interactions within the *csgD* 5′UTR, as well as changes in tertiary structure. Such interactions could potentially be discovered in a comparative analysis of sequences with single or double mutations as discussed earlier in the text.

We propose that our method is of particular value in studies of functional RNA modules, especially when two-mutation data sets are included. Additionally, this strategy will work well when adapted to many other aspects of gene expression and its control.

## SUPPLEMENTARY DATA

Supplementary Data are available at NAR Online: Supplementary Table 1 and Supplementary Figure 1.

## FUNDING

The Swedish Science Research Council (VR). Sequencing was performed by the SNP&SEQ Technology Platform in Uppsala. This platform is part of Science for Life Laboratory at Uppsala University and supported as a national infrastructure by the Swedish Research Council. Funding for open access charge: Swedish Science Research Council.

*Conflict of interest statement*. None declared.

## Supplementary Material

Supplementary Data
