# Supplementary material for: Massive functional mapping of a 5′-UTR by saturation mutagenesis, phenotypic sorting and deep sequencing
Source: Nucleic Acids Res. 2013 Apr 22;41(12):e122. doi: 10.1093/nar/gkt267 (PMC3695526; doi:10.1093/nar/gkt267)
Supplement: Supplementary Data [file supp_41_12_e122__index.html]

Massive functional mapping of a 5′-UTR by saturation mutagenesis, phenotypic sorting and deep sequencing — Massive functional mapping of a 5′-UTR by saturation mutagenesis, phenotypic sorting and deep sequencing — Supplementary Data 

# Massive functional mapping of a 5′-UTR by saturation mutagenesis, phenotypic sorting and deep sequencing

## Supplementary Data

files

**Files in this Data Supplement:**

- Supplementary Data - pdf file
