# Supplementary material for: Massive functional mapping of a 5′-UTR by saturation mutagenesis, phenotypic sorting and deep sequencing
Source: Nucleic Acids Res. 2013 Apr 22;41(12):e122. doi: 10.1093/nar/gkt267 (PMC3695526; doi:10.1093/nar/gkt267)
Supplement: Supplementary Data [file supp_gkt267_nar-00102-met-g-2013-File008.pdf]

Supplementary information for:

Massive functional mapping of a 5'UTR by saturation mutagenesis, phenotypic sorting and deep sequencing

Erik Holmqvist, Johan Reimegård and E. Gerhart H. Wagner

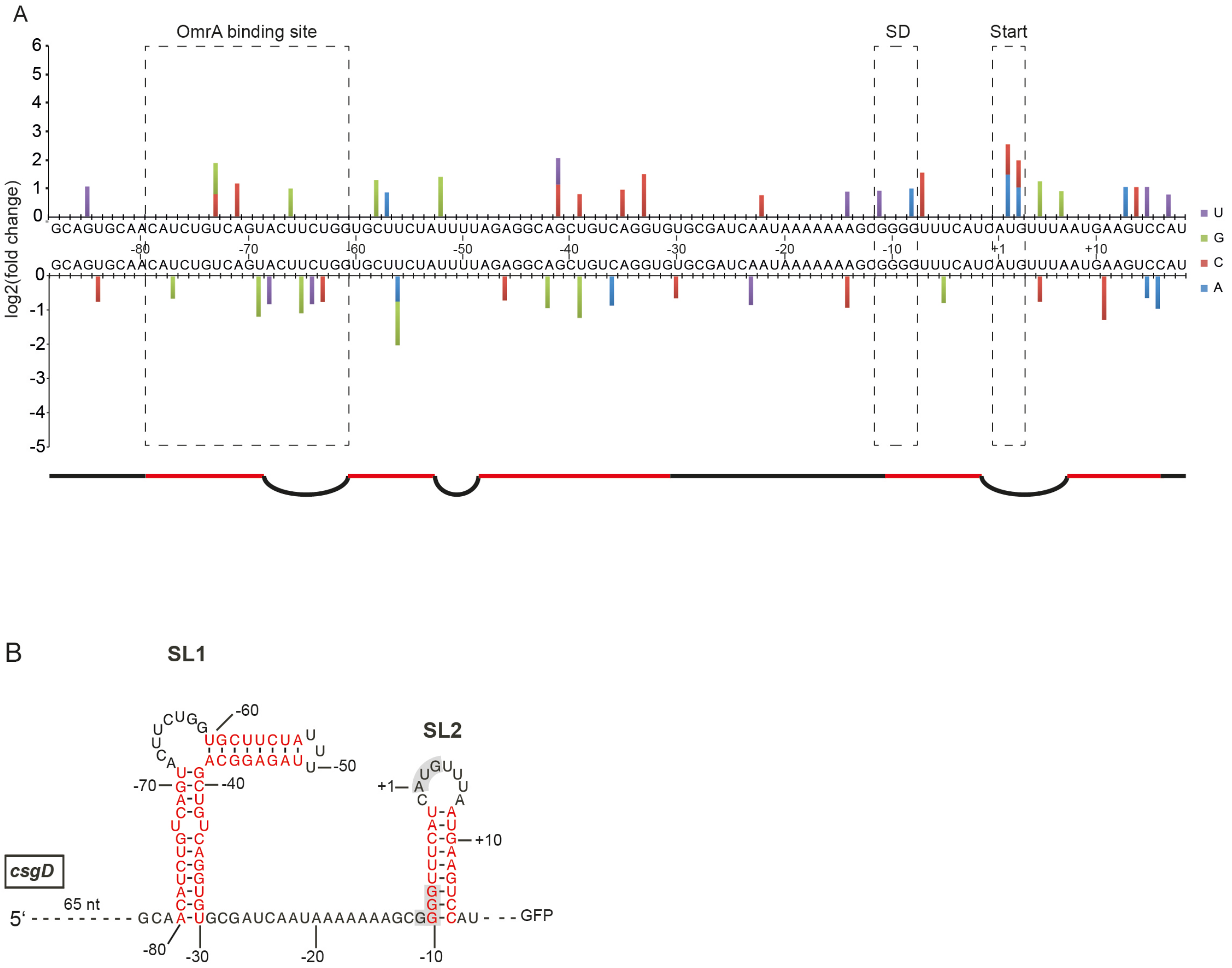

Supplementary Figure 1. Mutations associated with lowered *csgD-gfp* expression. (A) Significantly under- and overrepresented mutations in samples that were sorted for low fluorescence in the absence of OmrA compared to samples sorted for all fluorescence levels. Data were generated by Illumina sequencing. The RNA sequence of the *csgD* 5'UTR is shown in the middle of the graph in 5' to 3' direction. Numbers next to the sequence indicate nucleotide positions relative to A in the AUG start codon (set as +1). The OmrA binding site, the SD sequence, and the start codon are indicated with dashed boxes. A schematic under the graph represents the secondary structure of the *csgD* 5'UTR where black lines and bulges indicate unpaired nucleotides while red lines indicate paired nucleotides (same coloring as in (B)). (**B**) Sequence and secondary structure of the 5'UTR of *csgD* mRNA. Coloring and numbering as in (A).

Supplementary Table 1. Oligodeoxyribonucleotides used in this study.

Name	Sequence (5' to 3')	Used for
EHO-560	ccggagcgccgccagatatcgacgtctaagaaacc	pCsgD::GFP construction
EHO-561	ccggagcgccgcggcggatttgtcctact	pCsgD::GFP construction
EHO-570	ggccgaattccgctttccagtcgggaaacc	pOmrA construction
EHO-571	ggccggatccgacgggcttgtctgctcc	pOmrA construction
EHO-572	ggccggatccccacctgacgtctaagaaaccat	pOmrA construction
EHO-573	ggccgaattcagcgacagtaaattaggtgcgaa	pOmrA construction
EHO-522	cccatttagggctgatttattactaca	Error-prone PCR
EHO-575	caacaataatgtatgaccatgaatacta	Error-prone PCR
EHO-423	acatagtattcatggtcatacattattgttg	SDM: C+15A fwd
EHO-424	aaatagtattcatggtcatacattattgttg	SDM: C+15A, C+16A fwd
EHO-425	acttcattaaacatgatgaaacccc	SDM: rev for EHO-423 and EHO-424
EHO-628	cacagcagtgcaacatcagtcagtacttctggtgc	SDM: T-75A fwd
EHO-629	gcaccagaagtactgactgatgttgcactgctgtg	SDM: T-75A rev
EHO-630	cacagcagtgcaacatccgtcagtacttctggtgc	SDM: T-75C fwd
EHO-631	gcaccagaagtactgacggatgttgcactgctgtg	SDM: T-75C rev
EHO-632	cacacagcagtgcaacatctatcagtacttctggtg	SDM: -74 G:A fwd
EHO-633	caccagaagtactgatagatgttgcactgctgtgtg	SDM: -74 G:A rev
EHO-634	cacacagcagtgcaacatctttcagtacttctggtg	SDM: -74 G:T fwd
EHO-635	caccagaagtactgaaagatgttgcactgctgtgtg	SDM: -74 G:T rev
EHO-636	gtgcaacatctgtcagtacatctggtgcttctattttag	SDM: -66 T:A fwd
EHO-637	ctaaaatagaagcaccagatgtactgacagatgttgcac	SDM: -66 T:A rev
EHO-638	agcagtgcaacatctgtcagtacttatggtgcttctatt	SDM: -64 C:A fwd
EHO-639	aatagaagcaccataagtactgacagatgttgcactgct	SDM: -64 C:A rev
EHO-640	caacatctgtcagtacttcaggtgcttctattttagagg	SDM: -63 T:A fwd
EHO-641	cctctaaaatagaagcacctgaagtactgacagatgttg	SDM: -63 T:A rev
EHO-642	caacatctgtcagtacttctagtgcttctattttagaggca	SDM: -62 G:A fwd
EHO-643	tgcctctaaaatagaagcactagaagtactgacagatgttg	SDM: -62 G:A rev
EHO-644	caacatctgtcagtacttcttgtgcttctattttagaggca	SDM: -62 G:T fwd
EHO-645	tgcctctaaaatagaagcacaagaagtactgacagatgttg	SDM: -62 G:T rev
EHO-646	aacatctgtcagtacttctgatgcttctattttagaggcag	SDM: -61 G:A fwd
EHO-647	ctgcctctaaaatagaagcatcagaagtactgacagatgtt	SDM: -61 G:A rev
EHO-648	aacatctgtcagtacttctgttgcttctattttagaggcag	SDM: -61 G:T fwd
EHO-649	ctgcctctaaaatagaagcaacagaagtactgacagatgtt	SDM: -61 G:T rev
EHO-650	aacatctgtcagtacttctcgtgcttctattttagaggc	SDM: -62 G:C fwd
EHO-651	gcctctaaaatagaagcacgagaagtactgacagatgtt	SDM: -62 G:C rev
EHO-662	gtgtgcgatcaataaaaaaagcgaggtttcatcatgtttaatgaagt	SDM: -10 G:A fwd
EHO-663	acttcattaaacatgatgaaacctcgctttttttattgatcgcacac	SDM: -10 G:A rev
EHO-664	tgtgcgatcaataaaaaaagcggagtttcatcatgtttaatgaagtc	SDM: -9 G:A fwd
EHO-665	gacttcattaaacatgatgaaactccgctttttttattgatcgcaca	SDM: -9 G:A rev

SDM: Site-directed mutagenesis
